# Development of a Gold Nanoparticle Dispersion for Plasma Jet Printing on Solid Substrates

**DOI:** 10.3390/ma18122713

**Published:** 2025-06-09

**Authors:** Lan Kresnik, Peter Majerič, Darja Feizpour, Rebeka Rudolf

**Affiliations:** 1 Faculty of Mechanical Engineering, University of Maribor, 2000 Maribor, Slovenia; lan.kresnik1@um.si (L.K.); peter.majeric@um.si (P.M.); 2 Zlatarna Celje d.o.o., 3000 Celje, Slovenia; 3 Institute of Metals and Technology (IMT), 1000 Ljubljana, Slovenia; darja.feizpour@imt.si; 4 Pomurje Science and Innovation Center, 9000 Murska Sobota, Slovenia

**Keywords:** gold nanoparticles, USP, dispersion, plasma jet printing

## Abstract

Gold nanoparticles (AuNPs) were synthesised using ultrasonic spray pyrolysis (USP) with the addition of polyvinylpyrrolidone (PVP) as a stabilising agent and subsequently dried via lyophilisation. The resulting dried AuNPs were redispersed in ethanol and homogenised to ensure uniform dispersion. This AuNP dispersion was then deposited onto a ceramic substrate—aluminum oxide (Al_2_O_3_)—using plasma jet printing. Comprehensive characterisation of the dispersion, AuNPs, and the resulting printed lines was performed using the following methods: inductively coupled plasma optical emission spectroscopy (ICP-OES), scanning electron microscopy (SEM), transmission electron microscopy (TEM), selected area electron diffraction (SAED), scanning transmission electron microscopy (STEM), energy dispersive X-ray spectroscopy (EDS), ultraviolet-visible spectroscopy (UV-Vis), dynamic light scattering (DLS), measurements of dispersion viscosity and printed line roughness. ICP-OES confirmed consistent gold content in the AuNP dispersion, while the SEM and EDS analyses revealed predominantly spherical AuNPs with minimal aggregation and similar size distributions. TEM, SAED, and STEM/EDS confirmed that the crystalline structure and elemental composition of the AuNPs had diverse morphologies and strong gold signals. The UV-Vis, DLS, and zeta potential measurements indicated moderate colloidal stability, and thermogravimetric analysis (TGA) verified the AuNPs dispersion’s composition. The AuNP dispersion exhibited thixotropic behaviour favourable for printing applications, while confocal microscopy confirmed smooth, uniform printed traces, with an average surface line roughness of 1.65 µm. The successful use of plasma printing with the AuNP dispersion highlights its potential for functional material applications in electronics.

## 1. Introduction

The advancement of technology imposes stringent requirements on the materials and fabrication methods used in electronic components [1,2]. Printing techniques capable of creating a wide range of technologically important components have been of great interest recently. Conductive traces and metal electrodes are important parts of all electronic devices, even more so in areas such as space engineering. Satellites operate in harsh environments characterised by extreme temperatures, radiation exposure, and vacuum conditions, necessitating materials that ensure reliability and longevity [3,4]. Printed circuit boards (PCBs), integral to satellite electronics, demand conductive traces that can withstand these challenges [5,6,7]. Traditional fabrication methods and materials often fall short of meeting these demands, prompting the exploration of innovative solutions [8,9,10].

There are various printing techniques, including inkjet, aerosol jet, screen printing and 3D printing, that enable the deposition of metallic materials onto a substrate. However, nanoscale printing of metallic and conducting materials onto a substrate presents significant challenges [11,12,13]. One of the primary problems is ensuring strong adhesion between the printed materials and the substrate while preserving the substrate’s strength. Traditional printing techniques often require post-processing steps such as thermal annealing or thermal sintering. However, these high-temperature treatments can degrade the substrates, compromising their structural integrity. Beyond thermal limitations, achieving uniform and precise deposition on the inherently flexible and uneven surfaces of the substrates remains technically demanding [14]. Nanoscale dispersions must be formulated carefully to ensure stability, appropriate viscosity, and controlled drying characteristics while preventing clogging or inconsistencies during printing. This challenge is particularly pronounced in conventional methods such as inkjet, aerosol jet, and screen printing [15].

Plasma jet printing is emerging as an innovative alternative that addresses these challenges more effectively than conventional methods. By utilising a high electric field to eject printing materials and plasma onto substrates precisely, this technique ensures strong adhesion and accurate deposition while preserving the substrates’ structural integrity. Plasma jet printing is particularly advantageous for space applications, where traditional printing methods struggle, as it enables high-precision material deposition even under microgravity conditions [16,17,18].

The plasma-jet printer (PJP) is capable of operating in any orientation, including microgravity, because its material flow is driven by electromagnetic forces and the pressure of the carrier gas rather than gravity [19,20]. It works by atomising nanomaterial ink and using helium gas to transport the resulting mist to the printhead. The printhead itself contains two electrodes positioned around a dielectric ceramic tube and uses a high-voltage, low-frequency power source to create a dielectric barrier discharge plasma. As the nanomaterials pass through the atmospheric pressure plasma, they can be directed into precise patterns on a target surface. Importantly, the plasma jet also modifies the material properties during printing [21]. The ink is exposed to energetic particles like ions and electrons, which can cause the sintering of metallic nanoparticles during deposition. Plasma has been demonstrated to sinter metal nanoparticle films effectively, which offers a major advantage for in-space electronics manufacturing [22]. Unlike other printing methods that require additional heating or light-based annealing after material deposition, plasma-jet printing can perform both deposition and sintering in a single step. This streamlines the process, saving time and resources.

We selected plasma jet printing as the fabrication method due to several critical advantages it offers over conventional methods like inkjet or screen printing:

- Plasma jet printing inherently modifies the surface of the ceramic substrate through localised plasma treatment. This enhances surface energy and promotes strong adhesion of AuNPs—an essential factor when working with inert, non-porous substrates like ceramics [23];
- The plasma environment contributes to partial in situ sintering or activation of nanoparticles during deposition. This can reduce or even eliminate the need for high-temperature post-processing, which is especially valuable for maintaining the structural integrity of ceramic substrates or avoiding thermal mismatch issues [19,23];
- Unlike inkjet systems, which are sensitive to viscosity and prone to nozzle clogging when using nanoparticle dispersions, plasma jet systems are more robust and tolerant to a broader range of ink viscosities and particle sizes. This ensures reliable, consistent patterning without maintenance interruptions [11];
- Plasma jet printing supports fine feature resolution while maintaining a non-contact fabrication approach. This minimises mechanical damage and contamination risk on delicate or high-value substrates like technical ceramics [20].

While plasma jet printing offers a novel method for depositing metal nanoparticle inks, it comes with several limitations. One major constraint is that the equipment involved is complex and costly, requiring gas flow control, plasma generation hardware, and precise alignment between the nozzle and substrate—factors that can hinder its broader adoption compared to more straightforward techniques like inkjet printing [16]. Material compatibility also presents a challenge, as not all substrates or inks can endure plasma exposure; heat-sensitive or reactive materials may degrade or undergo undesirable interactions with the plasma plume. Furthermore, ensuring ink stability and printability under plasma conditions demands careful optimisation of colloidal properties. Lastly, while plasma jet printing is effective for prototyping and research, scaling it up for industrial-level production remains difficult due to issues with maintaining high throughput and consistent quality across large surface areas [18].

In modern electrical and communication systems, signal integrity is a crucial factor that determines performance and efficiency. However, as signals travel through conductors and transmission lines, they experience various forms of signal loss, which can degrade quality and limit effective transmission. Skin effect is one particularly significant phenomenon at high frequencies, which causes an alternating current (AC) to concentrate near the surface of a conductor rather than utilising its full cross-section. This effect increases the effective resistance of the conductor, leading to additional power dissipation and reduced efficiency. As frequencies rise, the impact of the skin effect becomes more pronounced, making it a critical consideration in radiofrequency (RF) circuits, high-speed data transmission and power distribution systems. Optimising trace geometry and reducing surface roughness is crucial for minimising the skin effect [24].

In the evolving landscape of printed circuit board (PCB) technology, conductive dispersions have emerged as a game-changing solution for creating electrical traces through innovative printing techniques. Unlike traditional copper etching methods, conductive dispersions enable additive manufacturing, allowing for flexible, lightweight and cost-effective circuit designs. By utilising materials such as silver, copper, carbon, and graphene, these dispersions facilitate the development of next-generation electronics, including flexible circuits, wearable devices, and rapid PCB prototyping [25,26,27].

Incorporating AuNPs into conductive dispersions offers significant advantages. Gold is renowned for its excellent electrical conductivity, resistance to oxidation, and chemical inertness, making it ideal for reliable conductive traces in PCBs [28,29]. The nanoscale size of AuNPs allows for lower sintering temperatures, enabling their use on temperature-sensitive substrates without compromising performance [30,31,32]. To prepare the dispersion for screen printing, various organic components, including binders, solvents, dispersants, and additives, are added to the AuNPs to create a dispersion, which is then used during the printing process [33].

The formulation of AuNPs-based dispersions involves several critical considerations. Stabilising the nanoparticles to prevent aggregation is essential for maintaining uniform dispersion within the product. The rheological properties must be optimised to ensure proper flow during the printing process, achieving the desired resolution and thickness of the printed traces. Additionally, the adhesion of the printed nanoparticles to the substrate is crucial for the mechanical stability of the printed patterns [34,35].

The aim of this research was to develop an AuNP dispersion tailored specifically for use with plasma jet printing on solid substrates. This study focuses on formulating a stable and functional dispersion with appropriate physical and chemical properties, such as particle size, viscosity and dispersion stability, that enable effective atomisation and transport within the plasma jet system. Additionally, this research seeks to demonstrate the successful deposition of the dispersion onto solid substrates while preserving or enhancing key material properties. The aim is to create a stable, functional AuNP dispersion that works well with PJP and can be used to print solid traces on ceramic substrates.

## 2. Materials and Methods

### 2.1. AuNP Synthesis

The AuNPs were synthesised on a proprietary Ultrasonic Spray Pyrolysis (USP) device [36]. The precursor for AuNP synthesis was hydrochloric acid HAuCl_4_ (hydrogen tetrachloroaurate (III) trihydrate; Glentham Life Sciences Ltd., Corsham, UK), dissolved in deionised water, with a concentration of 2 g/L HAuCl_4_ (an Au content of 50% gives a concentration of 1 g/L Au). The device uses four custom-made ultrasonic generators with 1.6 MHz piezo transducers (Liquifog II, Johnson Matthey Piezo Products GmbH, Redwitz an der Rodach, Germany) for generating aerosol droplets from the precursor solution. The ultrasonic generators are connected to four transport/reaction tubes made from quartz glass, with an internal diameter of 35 mm. Nitrogen gas, N_2_, was introduced inside the ultrasonic generators for transporting the aerosol droplets through the tubes at a flow rate of 6 L/min. The tubes are installed inside a reaction tube furnace with three heating zones, with a length of 500 mm for each heating zone. In the first heating zone, aerosol droplet evaporation is carried out at a temperature of 200 °C. A reaction gas, hydrogen, H_2_, is introduced after the first heating zone at a flow rate of 5 L/min. The two remaining heating zones operate at a temperature of 400 °C each for reactions between the dried aerosol particles with the hydrogen and the formation of dense AuNPs. The final AuNPs are collected in a gas-washing bottle collection system composed of three consecutively connected gas-washing bottles for each transport/reaction tube. The gas-washing bottles contain a collection medium, which is a solution of deionised water with a nanoparticle stabiliser, polyvinylpyrrolidone (PVP, average MW 50,000; Sigma-Aldrich, Darmstadt, Germany), with a concentration of 2.5 g/L. The synthesis time for a single batch of AuNP suspension was 5 h.

The USP parameters were selected from the results of extensive experimental work conducted on the custom-built, proprietary USP device [36]. The selected parameters yield a favourable AuNP suspension synthesis output in terms of quantity, sizes, and shapes of AuNPs, with an absence of agglomeration. Lower concentrations of PVP led to nanoparticle agglomeration and sedimentation, visible to the naked eye. Higher concentrations prolong the lyophilisation step due to increased water binding, reduced diffusivity, and slower sublimation. This can affect nanoparticle drying, redispersibility, and scalability [36].

The produced AuNPs’ suspensions were concentrated by rotary evaporation on a Büchi Rotavapor R-300 (Büchi Labortechnik AG, Flawil, Switzerland). The concentration parameters were evaporating flask rotation of 200 rpm, bath temperature 40 °C, and evaporation pump vacuum 24 mBar. The obtained suspensions were then concentrated further with a PTFE 100,000 MWCO filter membrane (0.1 μm pore size) using a Centurion C2000 centrifuge (Centurion Scientific Limited, Chichester, UK) at 10,000 rpm. The final concentration of AuNPs in the suspension was 5.04 mg/mL Au, analysed with ICP-OES before lyophilisation (freeze-drying).

The lyophilisation was performed on a Labfreez Instruments FD-200F (Labfreez Instruments Group Co., Ltd., Changsha, China). The freezing stage was carried out at −40 °C and ambient pressure for 6 h. The primary drying (sublimation) was then performed at a temperature of +20 °C and a pressure of 3 Pa for 12 h. The secondary drying followed, with a set temperature of +30 °C and a pressure of 3 Pa for 30 h. The total freeze-drying time was 48 h. The produced dried AuNPs were embedded in a PVP matrix, with a uniform porous structure and cake appearance.

### 2.2. Preparation of the AuNP Dispersion

The AuNP dispersion for printing was prepared using dried AuNPs stabilised with PVP, which were synthesised and lyophilised in Zlatarna Celje d.o.o., Celje, Slovenia.

A volume of 500 mL of ethanol (purity 99.8%, Sigma-Aldrich, Darmstadt, Germany) was measured and transferred into a glass beaker. The dried AuNPs were added to the ethanol gradually under continuous stirring. The mixture was stirred at room temperature at 200 rpm using a magnetic stirrer to facilitate the dispersion of the AuNPs. Stirring was continued for 120 min to achieve a homogeneous dispersion. Afterwards, the dispersion was poured into a glass flask and sealed to prevent evaporation of the solvent.

Additionally, a dispersion containing 25 ppm of dried AuNPs was prepared in deionised water for particle characterisation (UV-Vis, DLS, and zeta potential).

### 2.3. Plasma Jet Printing of AuNP Dispersion on Al_2_O_3_

**Figure 1:**
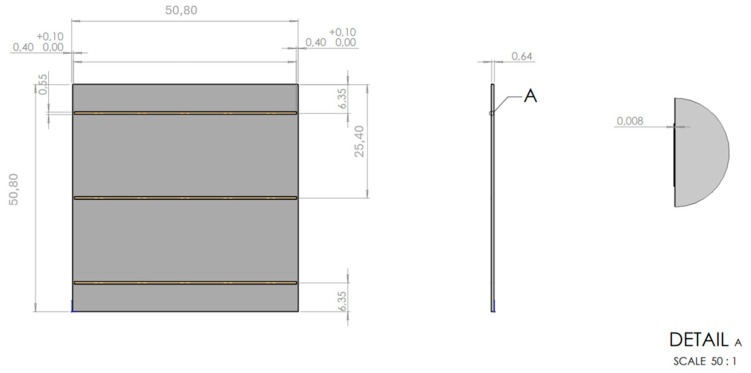
Sketch of our proposed PCB design with dimensions of each parameter.

The conductive lines were printed on a ceramic substrate, Al_2_O_3_ (96%), with a length and width of 50.8 mm and a thickness of 0.635 mm. Figure 1 shows the sketch of our proposed PCB. This sketch outlined the dimensions and structural features required for our experiment and was utilised directly as the template for the plasma jet printing stage.

The first printing trial (5 experiments) was carried out using an FG5001S generator equipped with a PFW10 nozzle (Plasmatreat GmbH, Steinhagen, Germany). The system was operated at a power range of 530–760 W, with nitrogen and air as the process gas, supplied at a flow rate of 33–50 L/min. The nozzle diameter was 5 mm, and the printing speed was maintained between 1 and 2.5 m/min. The precursor flow rate was set between 15 and 30 mL/h, while the carrier gas flow rate ranged from 0 to 15 L/min. The first 5 experiments were unsuccessful because the clogging at the jet head, particularly at the nozzle opening and feeding inlet, was encountered during the process. This problem interrupted the plasma flow and caused irregular material deposition, reducing the overall quality and reproducibility of the printed structures.

As a result, a second trial was carried out, where the AuNP dispersion was diluted with ethanol. The second printing was carried out using the same plasma system and jet nozzle as during the first trial. The power was set to 530 W, with a process gas flow rate of nitrogen (N_2_) at 33 L/min. The working distance (D) between the nozzle and the substrate was maintained at 5 mm. The deposition speed was controlled at 2.5 m/min. A precursor solution was injected directly into the plasma, with a flow rate of 3 mL/min. The number of deposition cycles was 4. Three traces were printed, with a length of 50 mm and a width of 0.55 mm. To ensure the formation of straight lines, the substrate was masked with adhesive tape, covering all areas except for the regions designated for printing.

### 2.4. Characterisation of the AuNP Dispersion and AuNPs

#### 2.4.1. Inductively Coupled Plasma Optical Emission Spectroscopy (ICP-OES)

The Au concentration in the AuNP dispersion post USP, and in the printing dispersion was measured using an Agilent 7500 CE ICP-OES spectrometer (Agilent, Santa Clara, CA, USA), operated at 1.5 kW with a Meinhard nebuliser. Plasma gas flow was 15 L/min; nebuliser flow, 0.85 L/min; make-up gas, 0.28 L/min; and reaction gas, 4.0 mL/min. Calibration was performed with matrix-matched standards. The relative measurement uncertainty was ±3%.

#### 2.4.2. Scanning Electron Microscopy (SEM) and Energy Dispersive X-Ray Spectroscopy (EDS)

The dried AuNPs and prepared ink were analysed with SEM and EDS, using a Sirion 400NC SEM (FEI, Hillsboro, OR, USA), equipped with an INCA 350 EDS (Oxford Instruments, Abingdon, UK). The samples were prepared on standard SEM holder stubs with conductive carbon adhesive tape. The prepared AuNP ink was diluted with ethanol, then put dropwise onto the SEM stub holder with carbon tape and left to dry in a desiccator, while the dried AuNPs were simply transferred to the holder with carbon tape before analysis. A total of 500 nanoparticles were measured for each sample for the reproduction of a size distribution chart and descriptive statistics from the measurements.

#### 2.4.3. Transmission Electron Microscopy (TEM) and Energy Dispersive X-Ray Spectroscopy (EDS)

The dried AuNPs embedded in a PVP matrix were mixed with deionised water to dissolve the PVP and release the nanoparticles. The resulting solution was drop-cast onto a TEM grid (lacey formvar carbon film, 300 mesh, Cu) and left to dry in a desiccator overnight before analysis. The sample was examined using imaging, electron diffraction, and elemental composition analysis with a Talos F200i (Thermo Fisher Scientific, Waltham, MA, USA) transmission electron microscope (TEM). The microscope was equipped with a 4k × 4k Ceta™ 16M camera (Thermo Fisher Scientific, Waltham, MA, USA) and an attached Bruker XFlash 6 | 30 energy-dispersive X-ray spectrometer (EDS) (Bruker, Billerica, MA, USA), featuring an energy resolution of 129 eV and a 30 mm^2^ active area chip for a large solid angle. The TEM was operated at an accelerating voltage of 200 kV. The scanning TEM (STEM)/EDS analyses were conducted under standard acquisition conditions.

#### 2.4.4. UV-Vis Spectroscopy

The absorbance spectra were collected using an Infinite 2000 Tecan i-control spectrophotometer using 4.5 mL disposable cuvettes (Thermo Fisher Scientific, Waltham, MA, USA). The absorbance spectra were collected from 350 to 800 nm, with a wavelength step size of 2 nm. Deionised water was used as a blank reference.

#### 2.4.5. Dynamic Light Scattering (DLS) and Zeta Potential

The DLS and zeta potential measurements of the AuNP dispersion were made on a Malvern Zetasizer Nano ZS instrument (Malvern Panalytical, Worcestershire, UK) equipped with Zetasizer Software version 7.13. An Omega cuvette, Anton Paar GmbH, Graz, Austria (Material No. 225288) was used. The measurements were carried out at a temperature of 25 °C. 

#### 2.4.6. Thermogravimetric Analysis (TGA)

Thermal stability was evaluated using a TGA/SDTA 851 analyser (Mettler Toledo, Columbus, OH, USA). Samples were heated from 25 °C to 500 °C in nitrogen (20 mL/min), then from 500 °C to 800 °C in oxygen, both at 10 K/min.

#### 2.4.7. Rheology Characteristics

Rheological properties were measured using a Haake Mars 60 rheometer (Thermo Fisher Scientific, Waltham, MA, USA) with a 60 mm cone-plate geometry at 25 °C. A 4 mL sample was tested under a shear protocol: 1 s^−1^ for 400 s, 200 s^−1^ for 100 s, and again 1 s^−1^ to assess recovery.

#### 2.4.8. Surface Roughness of the Printed Line

Surface roughness was measured on the AuNPs printed line using a confocal optical microscope (Axio CSM 700, Zeiss, Oberkochen, Germany). Measurements were taken at 1500× magnification (scan area ~117 × 93 µm). The arithmetic mean height (Sa) was evaluated at three locations for statistical reliability.

#### 2.4.9. Statistical Analysis

The descriptive statistics and data analysis were conducted using Microsoft Excel (Microsoft Corp., Redmond, WA, USA), utilising its built-in functions.

## 3. Results

### 3.1. Inductively Coupled Plasma Optical Emission Spectroscopy (ICP-OES)

The results of the Au concentration in the AuNP dispersion after USP and for printing are presented in Table 1.

### 3.2. Scanning Electron Microscopy (SEM) and Energy Dispersive X-Ray Spectroscopy Analyses (EDS) Results of AuNPs

**Figure 2:**
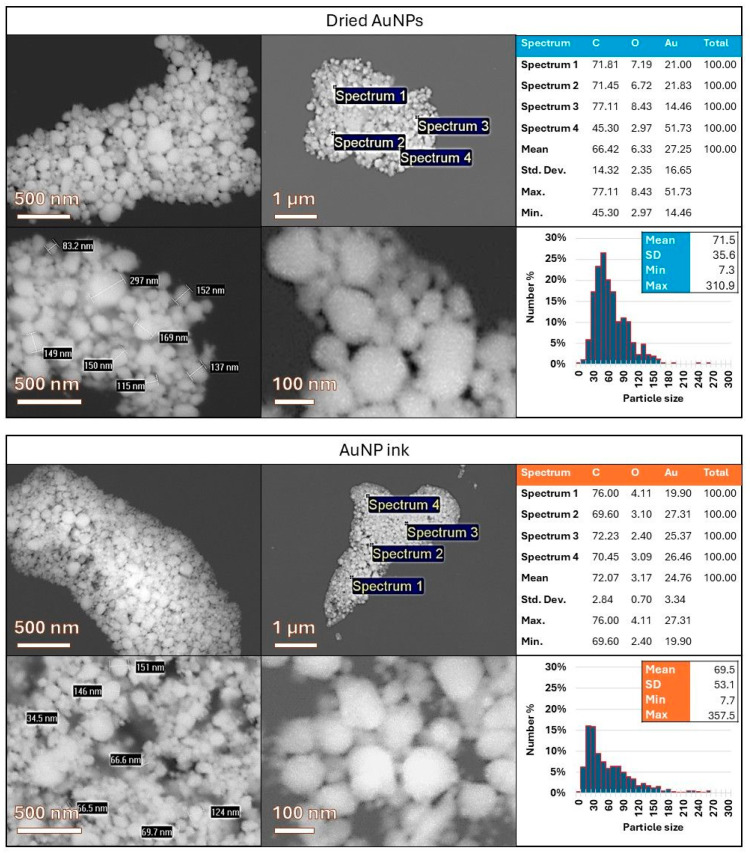
SEM and EDS results, with particle size measurements of the AuNPs in dried form and the prepared dispersion.

The dried AuNPs and the prepared AuNP dispersion were characterised with SEM and EDS for a direct comparison of the AuNPs’ shapes, sizes, and agglomeration states in these different dispersion formulations. Figure 2 shows the AuNPs analysed from the dried PVP cake and from the prepared dispersion. The shapes of the AuNPs were mostly spherical, with some irregularly shaped particles. The smaller AuNPs below 100 nm were predominantly spherical, while the irregular shapes were more present in larger AuNPs. The smallest measured AuNPs were around 10 nm in diameter, while the largest recorded were around 300 nm. The AuNPs were present as individual particles or in clusters, which may be the result of the presence of the PVP stabiliser, grouping the individual particles together during the preparation of the samples for SEM. Upon closer examination, the clusters contained single discrete particles, and aggregation or agglomeration was usually not present.

The EDS analysis showed a high value of carbon, C, because of the sample holder, as the samples were prepared on an SEM stub holder with carbon tape, as well as due to the presence of the stabiliser PVP. Oxygen, O, may also be attributed to the presence of the stabiliser PVP. Au was obtained in both sample types, confirming the AuNP content. The presence of the PVP stabiliser also hinders the SEM image acquisition of the samples somewhat, as it is a non-conductive compound that blurs the edges of the AuNPs in the obtained SEM images.

Figure 2 includes size distribution charts for the sample types with a bin width of 10 nm. The size measurements showed similar values for the dried AuNPs and for AuNPs in the prepared dispersion, with comparable minimum and maximum sizes of the AuNPs (7.3 and 7.7 nm for the minimum values, with 310.9 and 357.5 nm for the maximum values, for the dried AuNPs and AuNP dispersion, respectively). The mean values were also quite close (71.5 nm for the dried AuNPs and 69.5 nm for the AuNP dispersion), while the standard deviation was greater in the prepared dispersion (35.6 nm for the dried AuNPs and 53.1 nm for the AuNP dispersion). The size distribution charts also showed a broader size range for the prepared dispersion. This may be attributed to some aggregation of the smallest AuNPs because of the dispersion preparation and later dilution of this dispersion with ethanol for the preparation of the sample for SEM and EDS analysis. Additionally, the measurement sample of 500 particles may have some effect on the differences in the obtained size distributions.

The comparison of nanoparticles in the dried AuNPs and the prepared AuNP dispersion showed minimal differences in the particle characteristics. Some aggregation of the particles was present in the prepared AuNP dispersion, as compared to the dried AuNPs.

### 3.3. Transmission Electron Microscopy (TEM) and Selected Area Electron Diffraction (SAED), Scanning Transmission Electron Microscopy (STEM) and Energy Dispersive X-Ray Spectroscopy (EDS) Analyses of AuNPs

**Figure 3:**
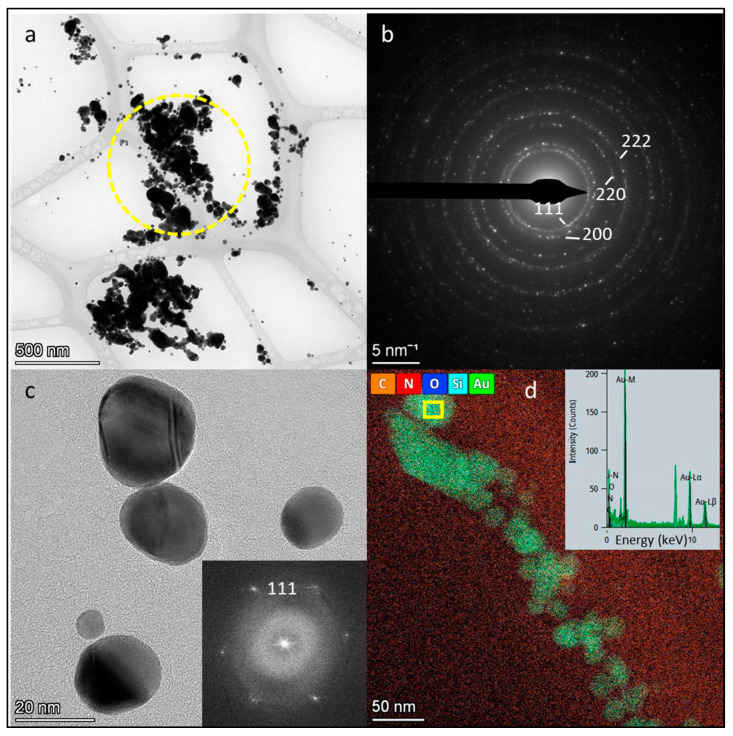
Bright-field (BF) TEM images of the dried AuNPs: (**a**) Low-magnification image with (**b**) selected area electron diffraction (SAED) pattern corresponding to the AuNPs marked by a yellow circle in (**a**). (**c**) High-magnification image of the AuNPs with an inset showing the 2D Fast Fourier Transform (FFT) of a selected area in the high-resolution BF TEM image. (**d**) Scanning TEM (STEM)/EDS mapping of the dried AuNPs, with an inset displaying the EDS spectrum of the particle marked by a yellow square. Acceleration voltage: 200 kV.

Figure 3 presents the TEM, selected area electron diffraction (SAED), and STEM/EDS results of the synthesised dried AuNPs. Figure 3a,c display AuNPs at low and high magnifications, revealing variations in size and shape. At lower magnification, some agglomeration is visible. The particle sizes range from 5 nm to 150 nm. The smallest AuNPs (~5–10 nm) are predominantly spherical, whereas the larger particles exhibit mainly rectangular shapes, with some also appearing hexagonal or triangular.

Figure 3b shows the SAED pattern, where diffraction rings and brighter spots—corresponding to larger particles—confirm the crystalline nature of the AuNPs. The diffraction spots align with the Au reflections (PDF No. 00-004-0784), as marked in the image.

Figure 3c provides a higher-resolution view, offering better insight into the morphology of the smaller AuNPs. Twinning effects associated with particle growth are also observed. The inset presents the 2D Fast Fourier Transform (FFT), with diffraction spots corresponding to the (111) plane.

Figure 3d illustrates the STEM/EDS analysis of the AuNPs. The inset shows strong EDS signals at Lα1 = 9.711 keV and Lα2 = 9.628 keV, confirming the presence of gold (highlighted in green in the mapping image). The peaks corresponding to carbon, nitrogen, oxygen, and silicon originated mainly from the TEM grid film and residual synthesis solvents. The unmarked peaks at Kα = 8.04 keV and Kβ = 8.91 keV are attributed to the TEM grid material.

### 3.4. UV-Vis Spectroscopy

**Figure 4:**
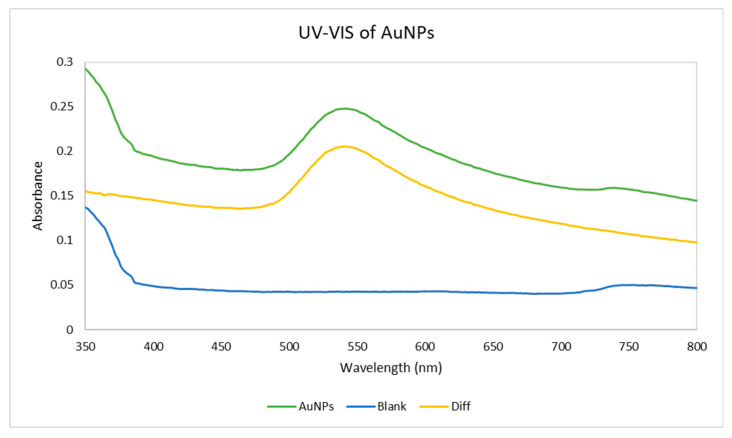
UV-Vis absorbance spectra of the AuNPs dispersed in deionised water, pure deionised water, and the difference between them.

The blue curve in Figure 4 represents the baseline absorbance of the deionised water, the green curve represents the absorbance of AuNPs in the deionised water, and the orange curve represents the difference between the blue and green curves, subtracting the blank values from the AuNPs’ suspension to isolate the contribution of the nanoparticles to the overall absorbance. The characteristic peak of AuNPs is generally observed between 500 and 600 nm due to the surface plasmon resonance effect [37]. A distinct peak in the 500–600 nm range can be observed on both the green and orange curves, indicating a successful formation of AuNPs. A comparison with the blue curve (blank) confirms the specific optical contribution of the nanoparticles.

### 3.5. Dynamic Light Scattering Results (DLS)

**Figure 5:**
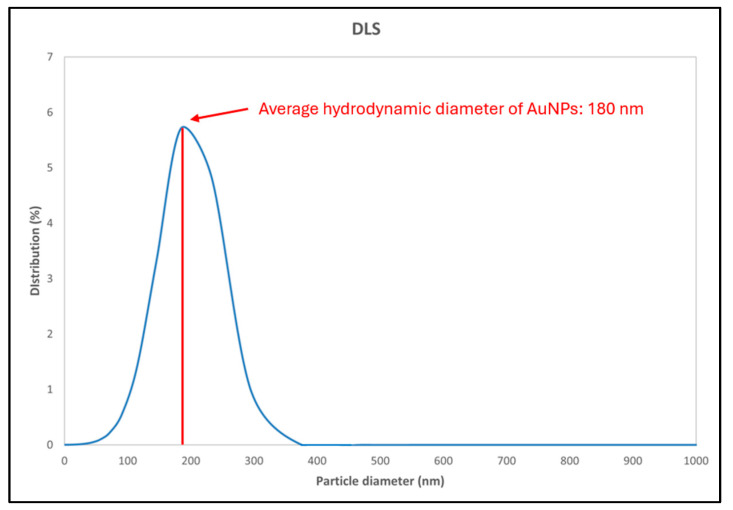
DLS measurement results showcasing the average particle diameter. Note: Reported DLS size is intensity-weighted, which may overestimate average diameter compared to number-weighted methods like SEM and TEM.

The purpose of the DLS analysis was to investigate the hydrodynamic diameter of the AuNPs and the polydispersity of the dispersion. Figure 5 shows the curve, which represents the hydrodynamic diameter of AuNPs in deionised water. The average hydrodynamic diameter is 180 nm, with a polydispersity of 25% and a Standard Deviation of 2.93 nm. The statistical analysis of the DLS measurement is presented in Table 2. The average particle diameters obtained from the DLS were consistently larger than those observed by TEM. This discrepancy arises because DLS measures the hydrodynamic diameter, which includes not only the solid particle core but also any surface coatings, bound solvent layers, and dynamic interactions with the surrounding medium. In contrast, TEM provides a direct visualisation of the particle core under dry conditions, excluding these additional contributions. Furthermore, DLS yields an intensity-weighted size distribution, making it particularly sensitive to the presence of larger particles or aggregates, whereas TEM analysis typically results in a number-weighted distribution. Since DLS intensity scales with the sixth power of diameter, a small population of larger particles or aggregates can disproportionately influence the average. Thus, the observed discrepancy is not unexpected. These methodological differences explain the larger apparent sizes observed in the DLS measurements.

### 3.6. Zeta Potential Results

**Figure 6:**
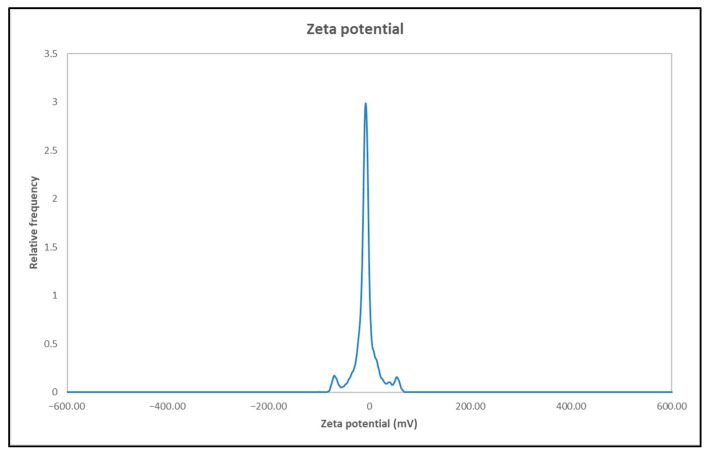
Zeta potential results of the AuNPs in deionised water.

The results of the zeta potential for the AuNP dispersion in deionised water are presented in Figure 6. The curve shows a peak at −12.4 mV. This relatively low negative value suggests moderate colloidal stability, indicating that electrostatic repulsion alone is insufficient to provide strong stabilisation. This can be attributed to the presence of PVP, which acts primarily as a steric stabiliser rather than an electrostatic one—PVP stabilises the AuNPs by forming a protective polymeric layer around them, preventing agglomeration through steric hindrance rather than charge repulsion [38]. 

### 3.7. Thermogravimetric Analysis Results (TGA)

**Figure 7:**
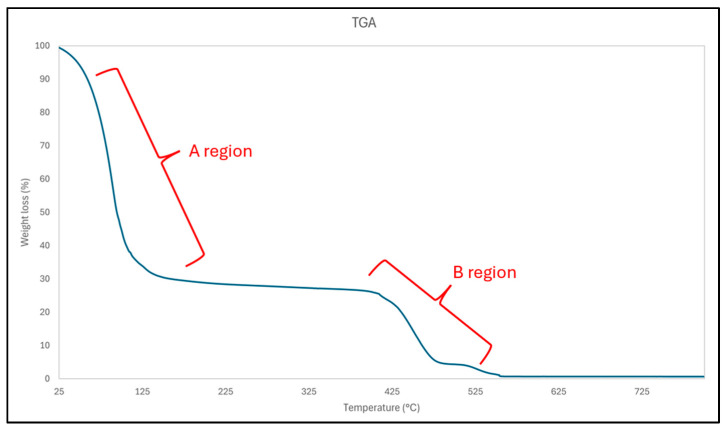
TGA curve of the AuNP dispersion.

Figure 7 shows the TGA curve of the AuNP dispersion, where its change in relative weight is visible as the temperature increases. The TGA curve shows an initial rapid mass loss up to approximately 120 °C (region A) due primarily to the evaporation of ethanol and any residual water present in the sample. This phase accounts for the most significant weight reduction. Between 150 and 400 °C, the mass loss occurs gradually, indicating the thermal decomposition of PVP. The degradation of the polymer stabiliser begins progressively around 250–350 °C. As the temperature increases to approximately 400–500 °C, a more pronounced mass loss is observed, corresponding to the complete decomposition of PVP and any remaining organic residues (region B) [39]. Above 500 °C, the mass stabilises, representing the inorganic content of the sample, primarily AuNPs, which remain intact at these temperatures.

### 3.8. Rheology Measurement

**Figure 8:**
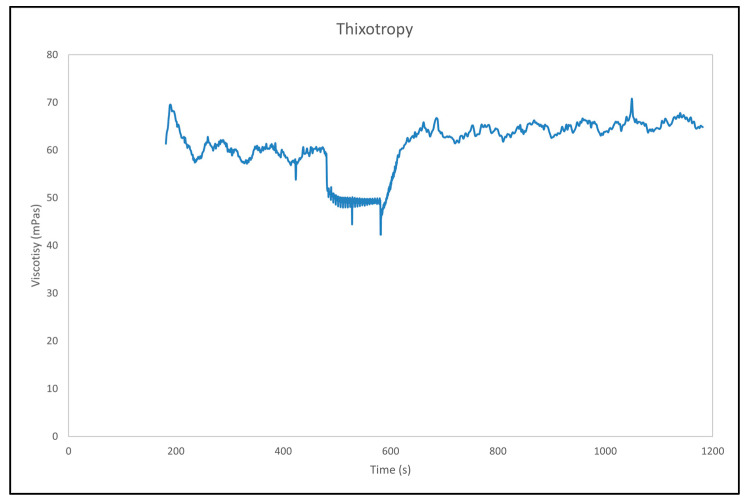
Thixotropy measurement of the AuNP dispersion.

Figure 8 illustrates the thixotropic behaviour of the AuNP dispersion, showing the evolution of viscosity as a function of time. The viscosity remains relatively constant during the initial stage, with only minor fluctuations, indicating the structural stability of the paste at rest. This is typical for thixotropic materials, where internal particle interactions maintain a stable network structure under quiescent conditions. At approximately 500 s, a noticeable decrease in viscosity is observed, corresponding to the application of an increased shear rate. This reduction is characteristic of thixotropic behaviour, where the internal structure of the material breaks down under shear, resulting in a lower viscosity [40]. Following the decrease in shear rate at around 600 s, the viscosity increases gradually and approaches its initial value. This recovery suggests the reformation of the internal particle network and partial restoration of the original microstructure. Subsequently, the viscosity stabilises again, indicating that the dispersion reaches a new equilibrium state.

### 3.9. Analysis of the Printed Lines

**Figure 9:**
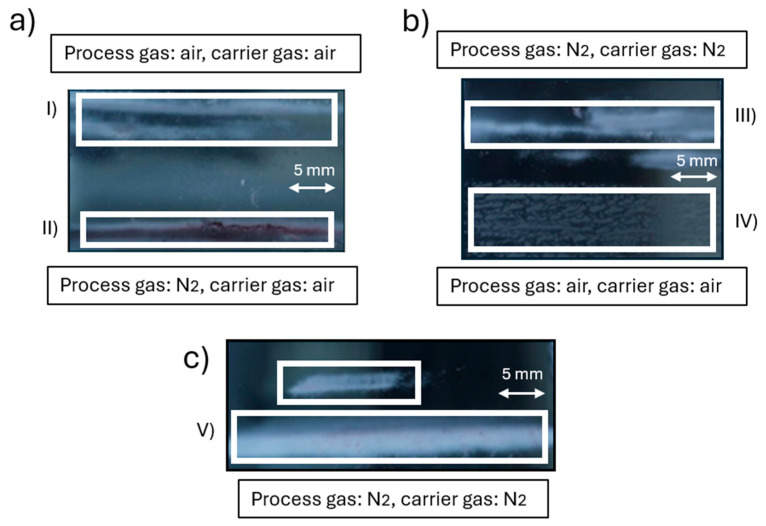
Unsuccessful plasma printing attempts under different combinations of process and carrier gases: (**a**) Sub-images I and II: Process gas = air, carrier gas = air (I); Process gas = N_2_, carrier gas = air (II). (**b**) Sub-images III and IV: Process gas = N_2_, carrier gas = N_2_ (III); Process gas = air, carrier gas = air (IV). (**c**) Sub-image V: Process gas = N_2_, carrier gas = N_2_.

Figure 9a–c illustrates representative examples (I–V) of unsuccessful plasma printing outcomes obtained using different combinations of process and carrier gases during the deposition of AuNP dispersion. Sub-images I and IV, both deposited under air as process and carrier gas, show poor adhesion and patchy morphology, likely due to limited nanoparticle sintering or surface charging. In sub-image II (N_2_ process gas, air carrier gas), a darker, irregular region is visible, potentially caused by partial nanoparticle aggregation or thermal effects at the substrate, but still lacking uniform film formation. Sub-images III and V, both utilising N_2_ as both process and carrier gas, reveal large pale or white deposits with poor definition. This white appearance is hypothesised to arise from residual PVP, which may not be fully decomposed under the lower reactivity of nitrogen plasma. In the absence of oxidative species (e.g., O_2_), PVP is less likely to break down, resulting in visible organic residue and non-conductive, poorly integrated films. These results emphasise the critical interplay between gas composition and organic surfactants in nanoparticle dispersions and the necessity of optimising plasma conditions to ensure the complete removal of stabilising ligands such as PVP.

**Figure 10:**
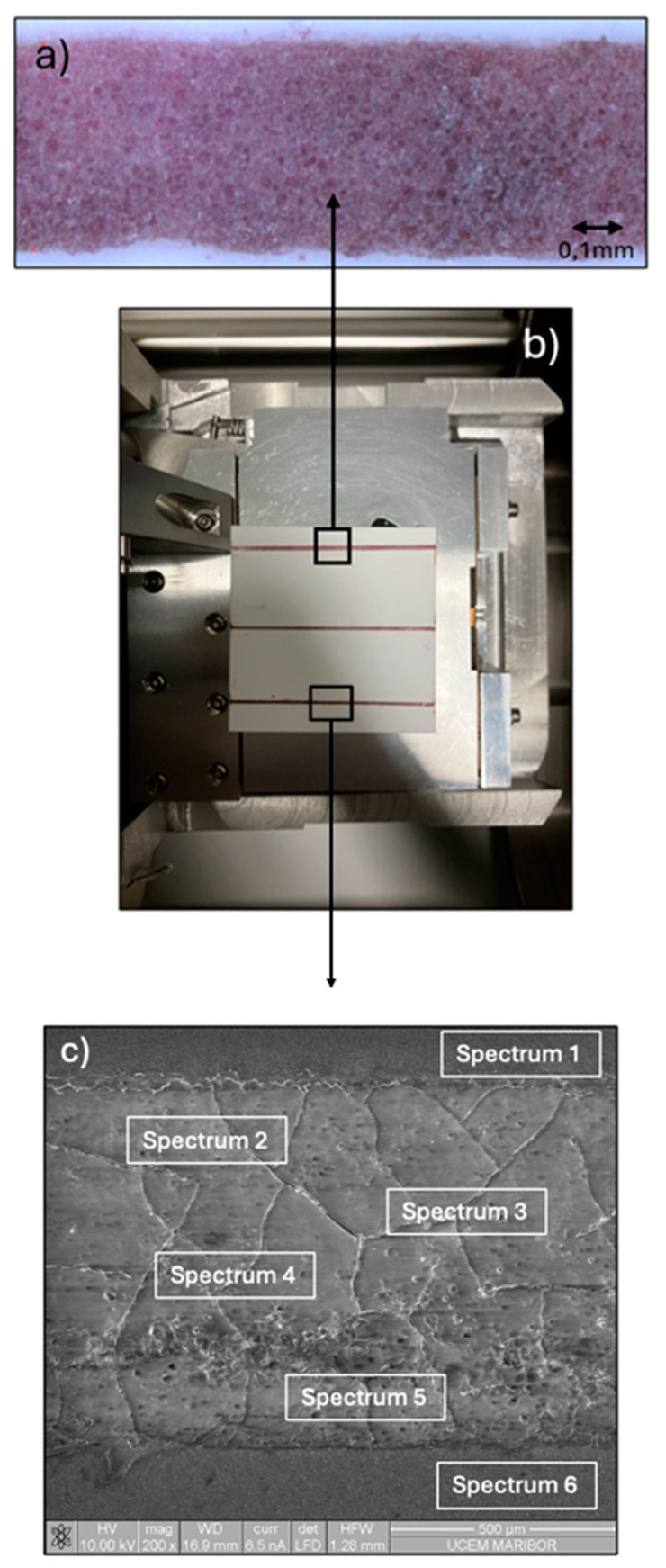
(**a**) a close-up of one printed line, (**b**) printed substrate, (**c**) SEM image of one line with visible areas for EDS analyses.

Figure 10b shows the printed substrate after the second plasma printing trial, where three distinct lines can be observed. Each line is 50 mm in length, 0.55 mm in width, and 0.008 mm in thickness. Figure 10a shows the magnified bottom line. Figure 10c shows the SEM image of the line. The presence of the purple colour confirms the successful incorporation of AuNPs along the printed line (Figure 10a). This indicates that there was no segregation, agglomeration, or formation of bulk gold—which would have been observed as a golden colour—thus confirming the nanostructured character of the deposited gold. A detailed examination of the printed line revealed that the edges were not straight, which poses a problem (the lateral roughness was too high compared to the requirements specified in Figure 1). Based on this, it will be necessary for future printing processes to design a mask that enables compliance with the tolerance requirements for edge straightness and lateral roughness, as required for these applications. This mask will also ensure that there is no outflow of dispersion during deposition on Al_2_O_3_. An EDS analysis was carried out to verify the elemental composition of the printed lines (Table 3). The EDS spectra revealed significant variations in elemental composition depending on the region analysed. Spectrum 1 and Spectrum 6 exhibited elevated carbon content, which is attributed primarily to the carbon sample holder used during the measurement. The presence of aluminium in these spectra, as well as the associated oxygen signal, is consistent with the Al_2_O_3_ substrate. Additionally, oxygen may also be attributed partially to the presence of the PVP used in the preparation process. Gold was detected in spectra 2–5, corresponding to areas where the AuNPs were printed on the substrate, confirming successful deposition.

**Figure 11:**
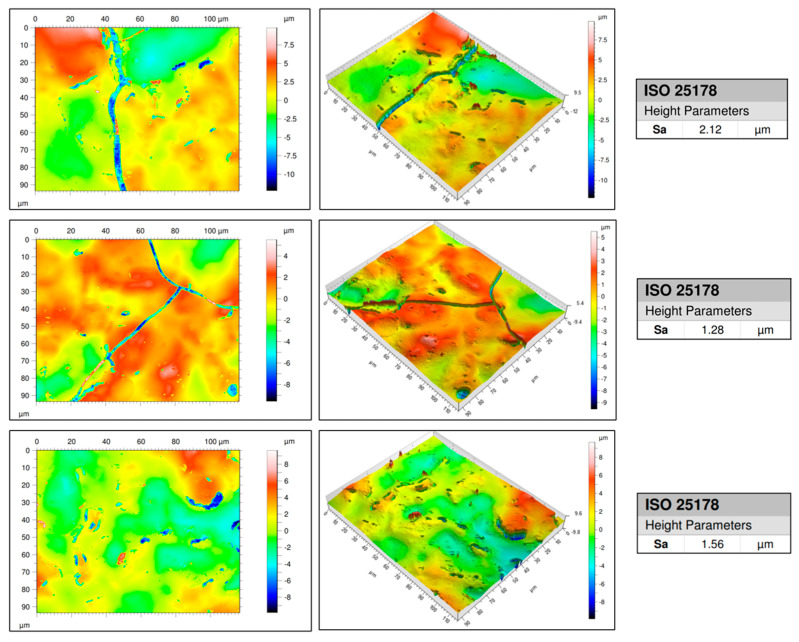
Surface roughness results showing the height profile (**left**), a 3D topographic image of the surface (**middle**), and the Sa values (**right**).

Figure 11 shows the height profile (left), a 3D topographic image of the surface (middle) and the Sa values (right). The Sa values across all three scan regions ranged from 1.28 to 2.12 µm, with an average Sa of 1.65 µm. These values confirm that the printed AuNP traces exhibit a sufficiently smooth surface topography. The absence of sharp peaks or significant waviness further suggests that the plasma printing process yields a stable and uniform film formation.

## 4. Discussion

The ICP-OES analysis provided quantitative data on the concentration of gold in both the dried AuNPs obtained after USP and the final dispersion for printing. This allowed for accurate monitoring of the gold content throughout the synthesis and redispersion processes, ensuring consistent nanoparticle yield and enabling comparison between different sample states.

The USP process has been used in a range of synthesis capacities, from laboratory to large-scale production [41,42]. The production yield depends on the USP parameters, such as gas and liquid flow rates through the reaction tube and the chemical composition of the precursor solution. Its main advantages are the commercial availability of the main process components (ultrasonic membranes, tube furnaces) and the continuous processing of materials. After initial investments, the continuous large-scale method provides for an economically suitable production process. As gold is an expensive raw material, the custom USP setup was used to produce AuNPs used in this investigation, which may be positioned as an intermediate level between laboratory and large-scale processes. The used USP device provides for a balance between lowering the initial raw material costs and providing for a suitable production yield required for obtaining the needed amount of AuNPs. Based on the possible demand for larger quantities of AuNPs, a full-scale industrial USP process may be designed to increase production due to its possibilities for scalability [43].

The comparative SEM and EDS analyses of dried AuNPs and the prepared AuNP dispersion provided valuable insights into the nanoparticle morphology, size distribution, and dispersion stability. In both sample types, the AuNPs were primarily spherical, with irregular shapes observed more commonly among the larger particles. This morphological similarity suggests that the synthesis and stabilisation process is robust and produces uniform nanoparticles consistently.

Both the dried and dispersed AuNPs displayed similar size distributions, with comparable minimum and maximum diameters and closely matching mean sizes. However, the broader size distribution in the dispersion, reflected by a higher Standard Deviation, may indicate minor aggregation during the redispersion and ethanol dilution steps. This suggests that while PVP stabilises the nanoparticles effectively, some degree of size variation is introduced during sample handling.

The EDS confirmed the presence of elemental gold in both sample types, while the high carbon and oxygen signals were attributed to the use of carbon tape and the PVP stabiliser. The non-conductive nature of PVP also affected the SEM imaging quality slightly by obscuring the nanoparticle edges, potentially impacting the agglomeration assessments.

The TEM, SAED, and STEM/EDS analyses confirmed the structural and elemental integrity of the synthesised AuNPs further. The TEM revealed a broad range of sizes (5–150 nm) and morphologies, including spherical, triangular, and polygonal shapes. The SAED and FFT confirmed the crystalline nature of the particles, particularly the presence of the (111) crystallographic plane. The EDS mapping supported the elemental composition, showing strong gold signals, with additional peaks corresponding to residual synthesis materials and the TEM grid.

The UV-Vis spectroscopy confirmed the successful formation of AuNPs, as evidenced by the surface plasmon resonance peak between 500 and 600 nm. The DLS analysis indicated a moderate polydispersity and a hydrodynamic diameter of around 180 nm. The zeta potential of −12.4 mV reflects the steric stabilisation effect of PVP, which supports colloidal stability to a moderate degree.

The TGA analysis identified ethanol, PVP, and gold as the major components of the dispersion. The final residual mass confirmed the gold content after the decomposition of organic materials, offering insight into the formulation’s stability and composition.

The rheological evaluation revealed thixotropic behaviour in the dispersion, suggesting reversible structural breakdown and recovery under shear. This is beneficial for applications requiring good flowability and shape retention, such as printing technologies.

The formulated AuNP dispersion demonstrated appropriate rheological and colloidal stability characteristics for use in plasma jet printing. This was evidenced by the successful deposition of three uniform lines, each approximately 5 cm in length, on Al_2_O_3_. The printed patterns exhibited good continuity and adhesion, indicating that the dispersion was well-suited for this additive manufacturing approach. Surface characterisation using confocal optical microscopy demonstrated low surface roughness and consistent deposition, with Sa values ranging from 1.28 µm to 2.12 µm. This indicates smooth and uniform film formation on ceramic substrates.

However, achieving an ultra-thin layer of approximately 8 µm presents challenges for SEM/EDX characterisation, which typically has a penetration depth of up to 6 µm. This limitation may result in incomplete analysis of the layer’s composition. Additionally, the presence of stabilisers containing elements such as oxygen and carbon can interfere with accurate elemental analysis. The EDS analysis (Table 3) revealed the presence of these stabilisers, suggesting the need for post-deposition thermal treatment to remove them.

The achieved low surface roughness (<2.8 μm) is particularly beneficial for high-frequency signal transmission (20–60 GHz), where the skin effect becomes significant [44,45]. A smooth surface minimises signal loss, which is crucial for the performance of printed electronic devices. The presence of stabilisers, while necessary for dispersion stability, must be managed carefully, as residual stabilisers can adversely affect the electrical properties of the printed layers. Thermal treatment has proven effective in reducing the stabiliser content, thereby enhancing conductivity.

The prepared AuNP dispersion has the potential to be used as a specialised conductive ink, especially for radio frequency applications [46]. One of the main benefits of plasma jet printing is that it allows for the direct use of dispersions without the need for extensive rheological optimisation, which is typically required for screen-printable or stencil-printable inks. In conventional processes, the inks must be engineered to meet strict viscosity and thixotropy criteria [47]. In contrast, plasma jet printing is a contactless, direct-write method that is far less dependent on the rheological properties of the ink or dispersion [23]. If the colloidal stability and particle size are appropriate for the nozzle and process, even low-viscosity dispersions can be printed effectively. This flexibility means that the synthesised AuNP dispersion—without additional thickening agents—can be used directly in plasma jet printing. Our study also suggests that utilising the dispersion directly after USP synthesis could significantly reduce the stabiliser content, potentially by up to five times. This approach may simplify the processing steps.

Overall, the combined characterisation confirms that the synthesised AuNPs possess desirable structural, chemical, and colloidal properties. Moreover, the successful application of plasma printing with the AuNP dispersion demonstrates the practical potential of this formulation for advanced functional material printing. The ability to directly print AuNPs using a plasma jet opens new possibilities for applications in printed electronics, including antennas, interconnects, biosensors, and microscale conductive traces on both rigid and flexible substrates. This technique is particularly attractive for applications requiring localised deposition on non-planar surfaces or low-temperature processing.

The main objective of this study was to determine the feasibility of printing our synthesised AuNPs using plasma jet techniques, with an emphasis on nanoparticle preparation, dispersion stability, and basic film formation. Electrical characterisation, although highly relevant for electronic applications, was not the primary focus of this initial phase. Future work will build on the findings presented here to evaluate the electrical properties of the printed structures in detail, including conductivity and resistivity measurements. These measurements will be essential for assessing the applicability of our approach in functional printed electronics. Further optimisation of the ink composition and plasma parameters will also be explored to enhance resolution and expand substrate compatibility. Ultimately, this approach could serve as a scalable platform for additive manufacturing of metal-based microelectronic components.

## 5. Conclusions

Based on our research findings, we draw the following conclusions:

- The synthesised AuNP dispersion is suitable for plasma jet printing, enabling the successful deposition of AuNP lines on Al_2_O_3_ with an achieved thickness of approximately 8 μm;
- The printed lines are purple in colour, confirming the presence of AuNPs; however, PVP remains present on the lines;
- The surface roughness of the printed lines ranged from 1.28 μm to 2.12 μm, thereby meeting the requirement for RF applications (<2.8 μm). This result demonstrates the potential for further development. The smoothness achieved is advantageous for applications in PCBs, particularly in high-frequency signal transmission, where surface quality is crucial.

## Figures and Tables

**Table 1:** ICP-OES analysis of the Au concentration after USP and in the AuNP dispersion.

Sample Name	mg/mL Au
AuNPs	2.81
AuNP dispersion	0.246

**Table 2:** Statistical analysis of the DLS measurements.

Results	AuNPs
Hydrodynamic diameter	180 nm
Polydispersity index	25%
Standard Deviation	2.93 nm

**Table 3:** EDS analysis of the printed line.

Spectrum	C	O	Al	Au
Spectrum 1	19.74	51.64	27.21	/
Spectrum 2	75.23	24.59	/	0.18
Spectrum 3	76.42	22.88	/	0.70
Spectrum 4	83.80	15.18	/	1.02
Spectrum 5	75.29	24.05	/	0.33
Spectrum 6	5.62	56.46	37.91	/

## Data Availability

The original contributions presented in this study are included in the article. Further inquiries can be directed to the corresponding author.

## Abbreviations

AuNPs—gold nanoparticles, Al_2_O_3_—aluminium oxide, DLS—dynamic light scattering, EDS—energy dispersive X-ray spectroscopy, ICP-OES—inductively coupled plasma optical emission spectroscopy, PVP—polyvinylpyrrolidone, SAED—selected area electron diffraction, SEM—scanning electron microscopy, TEM—transmission electron microscopy, TGA—thermogravimetric analysis, USP—ultrasonic spray pyrolysis, UV-Vis—ultraviolet–visible spectroscopy.
